# α-Galactosylceramide protects swine against influenza infection when administered as a vaccine adjuvant

**DOI:** 10.1038/srep23593

**Published:** 2016-03-23

**Authors:** Bianca L. Artiaga, Guan Yang, Timothy J. Hackmann, Qinfang Liu, Jürgen A. Richt, Shahram Salek-Ardakani, William L. Castleman, John A. Lednicky, John P. Driver

**Affiliations:** 1 Department of Animal Science, University of Florida, Gainesville, FL, USA; 2 Diagnostic Medicine and Pathobiology and Center of Excellence for Emerging and Zoonotic Animal Diseases (CEEZAD), College of Veterinary Medicine, Kansas State University, Manhattan, KS, USA; 3 Department of Pathology, Immunology, and Laboratory Medicine, University of Florida, Gainesville, FL, USA; 4 Department of Infectious Diseases and Pathology, University of Florida, Gainesville, FL, USA; 5 Department of Environmental and Global Health, University of Florida, Gainesville, FL, USA; 6 Emerging Pathogens Institute, University of Florida, Gainesville, FL, USA

## Abstract

Natural killer T (NKT) -cells activated with the glycolipid ligand α-galactosylceramide (α-GalCer) stimulate a wide array of immune responses with many promising immunotherapeutic applications, including the enhancement of vaccines against infectious diseases and cancer. In the current study, we evaluated whether α-GalCer generates protective immunity against a swine influenza (SI) virus infection when applied as an intramuscular vaccine adjuvant. Immunization of newly weaned piglets with UV-killed pandemic H1N1 A/California/04/2009 (kCA04) SI virus and α-GalCer induced high titers of anti-hemagglutinin antibodies and generated virus-specific T cells that localized in intrapulmonary airways and in alveolar walls. Vaccination with α-GalCer resulted in a systemic increase in NKT-cell concentrations, including in the respiratory tract, which was associated with complete inhibition of viral replication in the upper and lower respiratory tract and much reduced viral shedding. These results indicate that NKT-cell agonists could be used to improve swine vaccine formulations in order to reduce the clinical signs of SI infection and limit the spread of influenza viruses amongst commercial pigs.

Swine influenza (SI) is an important infectious disease of pigs caused by influenza A viruses (IAV)^1^. Some of these are capable of causing human pandemics. For example, the 2009 pandemic H1N1 virus (H1N1pdm09) caused thousands of deaths, millions of hospitalizations and led to billions of dollars in lost revenue for the pork industry. Although swine influenza (SI) is typically caused by only three subtypes of IAV (H1N1, H1N2, and H3N2), these continue to evolve at an ever-increasing pace. Addressing this threat has proven very difficult because currently available SI vaccines fail to provide sterilizing immunity, even when closely matched to viruses in the field^2^,^3^,^4^,^5^. Thus, there is an urgent need to explore new solutions to improve vaccinations against IAV infections in swine. One promising approach is the use of natural killer T (NKT) cells that may have potential to enhance vaccine responses when activated using synthetic glycolipids.

Invariant NKT-cells are a minor lymphocyte subset that share phenotypic characteristics of both NK cells and T lymphocytes and express a semi-invariant T cell receptor (TCR) repertoire that recognizes self and foreign glycolipid antigens presented by the non-polymorphic CD1d molecule. Often referred to as the “Swiss Army knife” of the immune system for their ability to stimulate diverse immune functions^6^, NKT-cells promote antimicrobial and antitumor responses through a combination of rapid release of cytokines^7^, maturing dendritic cells (DCs)^8^, activating NK cells^9^,^10^ and boosting polyclonal antibody production^11^,^12^. They also induce Th1-biased cellular responses that optimize host immune defenses against viral pathogens^13^, which underlies why mice genetically lacking NKT-cells are more susceptible to several viral pathogens including influenza viruses^14^,^15^,^16^,^17^.

NKT-cell agonists have been used as vaccine adjuvants in rodent models^18^. The glycolipid antigen most studied for this purpose is α-galactosylceramide (α-GalCer). It potently stimulates NKT-cells to release large quantities of cytokines that induce the *trans*-activation of a wide array of bystander cells including NK cells, DCs, and conventional T cells subsets [reviewed in^19^]. These NKT-cell-mediated responses boost the efficacy of influenza vaccines resulting in strong protection against lethal challenges with H1N1 or H3N2 virus strains in mouse models^20^,^21^,^22^,^23^,^24^. We previously investigated whether NKT-cell agonists also have potential for enhancing vaccine responses in swine and showed that pigs co-injected with α-GalCer and the model antigen hen-egg lysozyme (HEL) generate strong anti-HEL cell and antibody immune responses^25^.

In the current work we demonstrate the feasibility of using NKT-cell agonists to generate cellular and humoral immune responses to improve influenza vaccines in swine that are the natural host of SI viruses. These results indicate a potential veterinary use for NKT-cell adjuvants for protecting commercial swine from influenza viruses and other important pathogens.

## Results

### α-GalCer increases NKT cells in blood, lung, and lymphoid tissues

To investigate whether co-immunization with α-GalCer and inactivated CA04 virus protects swine from SI virus infection when injected into the neck muscle, where most swine vaccines are delivered, 3-week-old piglets were vaccinated with 10^6^ TCID_50_ UV-killed CA04 (kCA04) alone or with 100 μg/kg α-GalCer, or mock vaccinated with virus-free media. All groups contained pigs with a similar range of pre-trial NKT-cell frequencies (Table 1). Immunizations were repeated 16 days later, and pigs were mock inoculated or challenged intratracheally with 10^6^ TCID_50_ CA04 after 16 more days. Half of the piglets in each group were euthanized at 3 or 7 days post infection (p.i.), respectively. No differences were detected amongst any of the groups for body weight gain throughout the vaccination and challenge periods of the study (Table 1). No signs of discomfort or distress were observed for pigs injected with kCA04 or α-GalCer.

**Figure 1:**
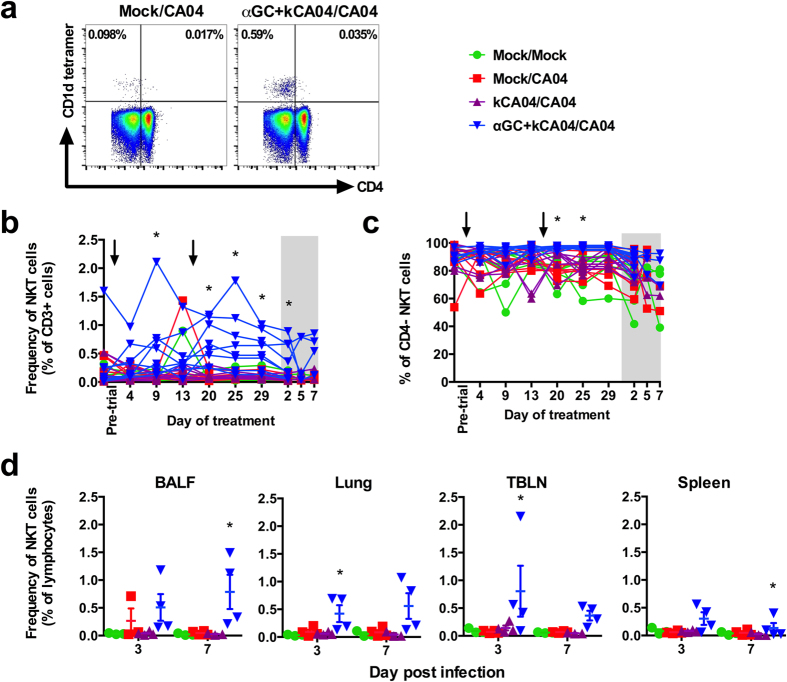
α-GalCer expands porcine NKT-cells that are mostly CD4^−^. (**a**) Representative flow cytometry plots comparing peripheral blood (PB) NKT-cells from a mock-vaccinated pig versus a pig vaccinated with α-GalCer. Single cell suspensions were membrane-labeled with anti-CD3 mAb and PBS57-loaded CD1d tetramer. Gating to discriminate CD4^+^ and CD4^−^ NKT-cell subsets was based on CD4 staining after gating on CD3^+^ lymphocytes. (**b**) Frequency of NKT-cells as a percentage of CD3^+^ lymphocytes in PB of individual pigs during the vaccination period and post infection (shaded region). (**c**) Percentage of CD4^−^ NKT-cells in PB during the vaccination period and post infection (shaded region). (**d**) Frequency of NKT-cells as a percentage of total lymphocytes in bronchoalveolar lavage fluid (BALF), lung, tracheobronchial lymph node (TBLN) and spleen at 3 and 7 days p.i. Differences in PB NKT-cell frequencies and subsets were analyzed using the SAS PROC MIXED procedure and the Turkey’s test was used to examine treatment differences at each time point for each dependent variable. Changes in BALF, lung, TBLN and spleen NKT-cell frequencies were analyzed using the Kruskal-Wallis test. *P < 0.05. Data are represented as mean ± SEM. Arrows indicate when vaccinations were administered.

To assess how α-GalCer affects the expansion and distribution of NKT-cells, pig cells were analyzed by flow cytometry during the vaccination and challenge periods using a mouse CD1d tetramer reagent that binds the porcine invariant NKT-cell TCR (Fig. 1a)^25^. Peripheral blood NKT-cell frequencies rapidly increased between 4 to 9 days after primary vaccination with α-GalCer and kCA04 and then decreased by day 13 (Fig. 1b). A similar change in NKT-cell frequencies was observed after the booster vaccination with the exception that maximum NKT-cell expansion occurred at day 4 (20 days after primary vaccination) rather than day 9 after α-GalCer co-immunization. NKT-cells remained high in αGC+kCA04/CA04 pigs during the 7-day post inoculation period (Fig. 1b). NKT-cell frequencies in all the other treatment groups remained low and stable during the entire trial. NKT-cell subsets that are functionally distinct in other species were analyzed according to membrane bound CD4^26^,^27^,^28^. Pigs contain few CD4^+^ NKT-cells in PB compared to mice and humans^25^. We found that α-GalCer administration expanded mostly the CD4^−^ subset (Fig. 1c).

The α-GalCer vaccination protocol primed an immune response that increased NKT-cell concentrations in BALF, lung tissue, TBLN and spleen after challenge (Fig. 1d). No change in NKT-cell frequency was detected in pigs mock vaccinated or vaccinated with kCA04 alone. To determine whether other leukocyte populations were affected by exposure to CA04 or α-GalCer, PB and tissues were compared by flow cytometry at the same time that they were analyzed for NKT-cells. No differences were found amongst any groups for conventional, regulatory and γδ T lymphocytes, B lymphocytes, monocytes, and granulocytes (Supplemental Fig. 2 and Supplemental Tables 1–4). Our observations demonstrate that α-GalCer injected into the neck muscle induces the increase of NKT-cells systemically, including in the respiratory tract where they were detected at high concentrations at 3 weeks after vaccination.

### Co-immunization of inactivated CA04 and α-GalCer boosts CA04-specific antibody responses

**Figure 2:**
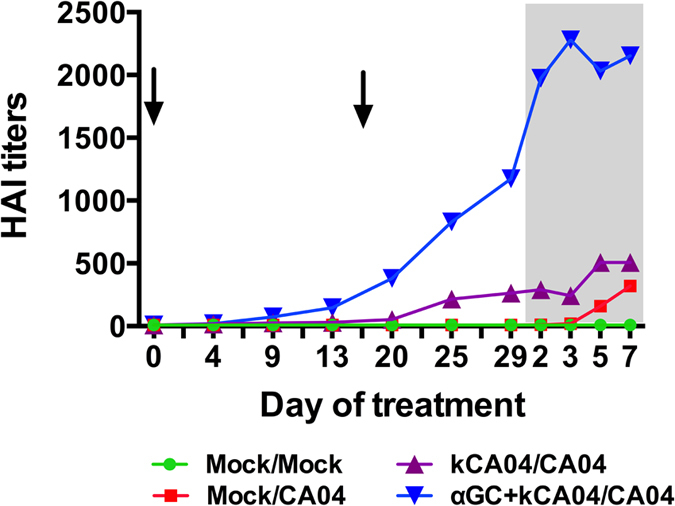
Geometric mean of HAI titers against CA04 H1N1 antigen in plasma at during the 32 day vaccination period as well as 2, 3, 5 and 7 days post-infection (indicated by the shaded region). Arrows indicate when vaccinations were administered. Geometric mean was calculated as the ‘n’th root product of ‘n’ numbers.

α-GalCer-activated NKT-cells can partially substitute for CD4^+^ T cell help to induce antibody production by B cells^12^,^29^,^30^. Therefore, we examined PB and BALF for SI virus-specific humoral responses during the course of the experiment. Prior to the start of the study all pigs were serologically negative for H1N1 SI virus antibodies by hemagglutination inhibition (HAI) assay. Only vaccinated pigs seroconverted to become H1N1 positive during the vaccination period. Average titers in plasma were significantly higher for pigs co-immunized with α-GalCer and kCA04 than kCA04 alone (Fig. 2). The adjuvant effects of α-GalCer induced CA04-specific antibodies from 13 days after primary vaccination with kCA04, which was 3 days before the booster vaccination. In contrast, CA04-specific antibodies were detected 25 days after primary vaccination in pigs that received kCA04 alone, which was 9 days after the booster vaccination. Peak antibody titers were measured between 2 and 3 days p.i. for α-GalCer treated pigs compared to day 5 p.i. for pigs vaccinated with virus alone. Low antibody titers were detected in mock-vaccinated pigs from day 5 p.i.

To evaluate the development of protective antibody responses in the lung mucosa, BALF was analyzed for levels of CA04-specific anti-hemagglutinin antibodies. Only BALF from the α-GalCer treated pigs contained detectable antibody titers, although at levels much lower than measured in plasma because BALF was diluted by lavaging the left lung with 50 ml of MEM (Table 2). These collective data indicate that the adjuvanticity of α-GalCer enhances both systemic and mucosal concentrations of influenza-specific antibodies.

### Vaccination with α-GalCer induces virus-specific cellular immunity that is variable amongst pigs

**Figure 3:**
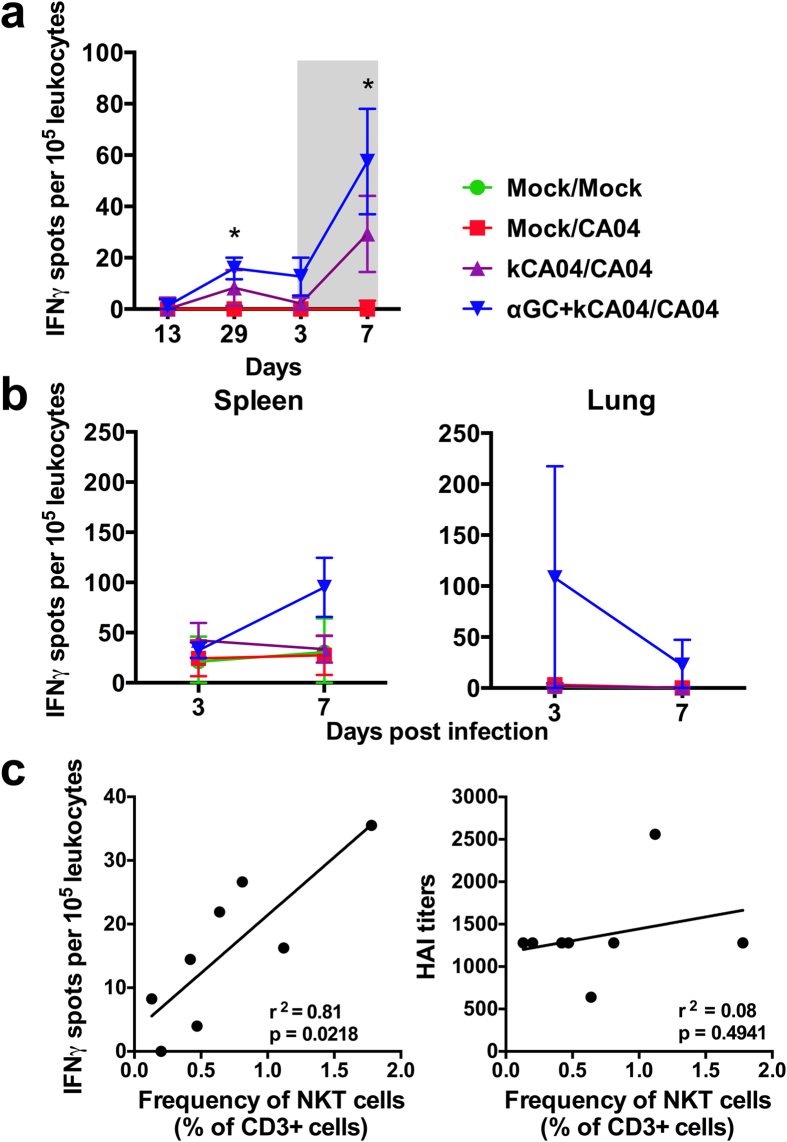
Cellular responses to CA04 H1N1 antigen were measured by IFN-γ-ELISPOT assays. IFN-γ production by CA04-reactive cells in (**a**) PBMCs during the vaccination period and post infection (shaded region) and (**b**) spleen and lung at 3 and 7 days p.i. Single cell suspensions were incubated for 72 h with UV-inactivated CA04 H1N1 virus. Results represent mean IFN-γ spots per 10^4^ leukocytes after subtracting spots counted in unstimulated wells. Results are displayed as the mean ± SEM. Means were compared using the Kruskal-Wallis test. *P < 0.05. (**c**) Correlations between peripheral blood NKT-cell frequencies and concentrations of CA04-reactive cells in PBMCs (left panel) and CA04-specific antibody titers in plasma (right panel) at day 29 after primary vaccination. Correlations were determined using linear regression analysis and the line in each plot represents the best linear fit.

Combined NKT-cell activation and influenza virus vaccination is known to boost vaccine-specific T cell immunity^20^,^21^,^22^,^23^, in part, by potently activating professional antigen presenting cells through cytokine secretion and CD40-CD40L interactions. Thus, we analyzed the frequency of CA04-reactive T cells within different tissues by IFN-γ ELISPOT assay, which involved re-stimulating single cell suspensions with kCA04 in culture for 72 h. α-GalCer induced low levels of CA04-reactive PBMCs during the vaccination period that increased at 7 days post challenge, although there was no significant difference compared to pigs vaccinated with kCA04 alone (Fig. 3a). α-GalCer-vaccinated pigs tended to have higher concentrations of CA04-reactive cells in spleen at 7 days p.i. and in lung at 3 and 7 days p.i. compared to pigs vaccinated with kCA04 alone (Fig. 3b). There was no statistical difference between groups for spleen and lung due to the large range of NKT-cell concentrations amongst the pigs enrolled in the α-GalCer treatment group, which may have contributed to the highly variable T cell responses to NKT-cell activation that we observed. Indeed, concentrations of CA04-reactive PBMCs were tightly correlated to the frequency of peripheral NKT-cells in pigs vaccinated with α-GalCer (r^2^ = 0.81, p-value = 0.02) (Fig 3c; left panel). No correlations were found between NKT cells and CA04-reactive PBMCs for pigs that were vaccinated with kCA04 alone (data not shown). Collectively, these data suggests that a certain threshold level of NKT-cells is needed for α-GalCer to induce effective virus-specific cellular immunity. In contrast, no correlation was detected between NKT-cell concentrations and anti-hemagglutinin antibody titers (r^2^ = 0.08, p-value = 0.49) (Fig 3c; right panel), indicating that NKT-cell-mediated humoral immunity may not depend on whether pigs contain high or low levels of NKT-cells.

### Localization of CD3^+^ T cells after SI virus infection

**Figure 4:**
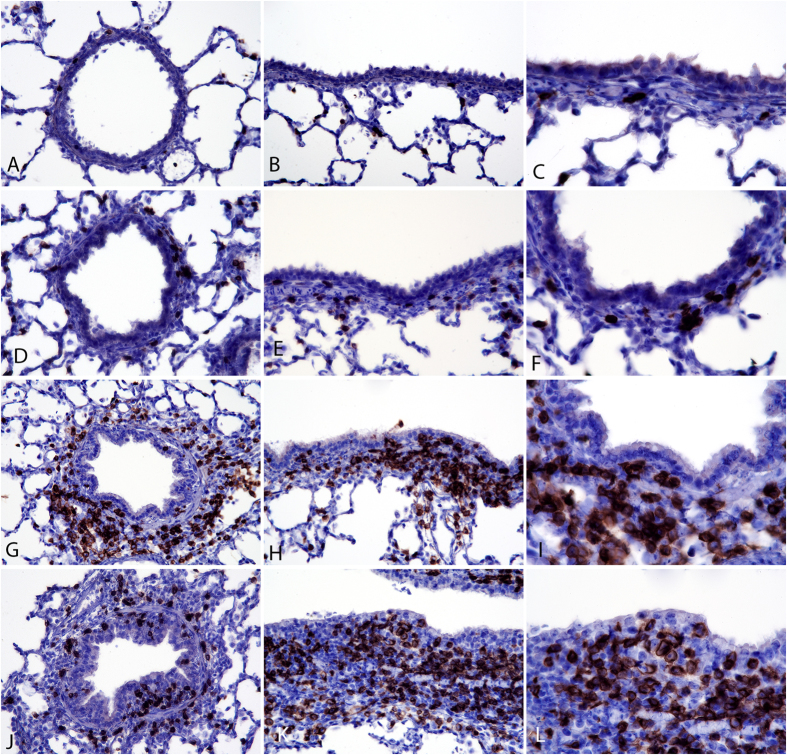
Localization of CD3^+^ T cells after SI virus infection. Mock/Mock: Images (**A–C**). Images (**A,B**) are transverse and longitudinal sections of CD3 stained bronchioles at 180× magnification. Image (**C**) is at 360×. Mock/CA04: Images (**D–F**). Images (**D,E**) are transverse and longitudinal sections of CD3 stained bronchioles at 180× with low density CD3^+^ T-cells in bronchiolar walls. Image (**F**) is at 360× showing dark brown, CD3-stained T-cells in bronchiolar walls. kCA04/CA04: Images (**G–I**). Images (**G,H**) are transverse and longitudinal sections of CD3 stained bronchioles at 180× with thickened bronchiolar walls containing moderate to high density aggregates of CD3^+^ T-cells. Image (**I**) is at 360× showing high density CD3^+^ cells in the bronchiolar wall below columnar to cuboidal bronchiolar epithelial cells. αGC+kCA04/CA04: Images (**J–L**). Images (**J,K**) are transverse and longitudinal sections of CD3 stained bronchioles at 180× with markedly thickened bronchiolar walls containing moderate to high density aggregates of CD3^+^ T-cells with infiltration of CD3^+^ T-cells within bronchiolar epithelium which is hyperplastic. Image (**L**) is at 360× showing high density CD3^+^ cells in the bronchiolar wall within hyperplastic cuboidal bronchiolar epithelial cells.

Lung tissue was examined by immunohistochemistry to evaluate the effects of vaccination and infection on T cell recruitment and distribution in the respiratory tract. Virus infection induced localization of CD3^+^ T cells in bronchial, bronchiolar and alveolar walls. CD3^+^ T cells were most densely localized in bronchiolar walls of virus-inoculated pigs. In Mock/Mock pigs, only rare CD3^+^ cells were visualized in the lamina propria of bronchioles (Fig. 4A–C). Virus inoculation alone (Mock/CA04) resulted in low-density infiltrates of CD3^+^ cells multifocally in bronchiolar walls at 3 days p.i. (Fig. 4D–F). In contrast, in both groups of vaccinated pigs (with and without α-GalCer), high numbers of CD3^+^ cells could be readily visualized around bronchioles in lamina propria and adventitia. Few intra-epithelial CD3^+^ cells were seen in the vaccinated and challenged group (kCA04/CA04) (Fig. 4G–I). Pigs receiving α-GalCer (αGC+kCA04/CA04) had higher density of intra-epithelial CD3^+^ cells associated with hyperplastic bronchiolar epithelium than did vaccinated pigs that did not receive α-GalCer (Fig. 4G–I versus Fig 4J–L). High-density CD3^+^ T cell infiltrates were present in bronchioles from pigs in both vaccinated groups at 7 days after virus inoculation compared to low density CD3^+^ cells in pigs at 7 days that only received virus (data not shown). Again, pigs receiving α-GalCer appeared to have higher density of CD3^+^ cells in their bronchiolar walls at 7 days than did vaccinated pigs that did not receive α-GalCer (data not shown).

### Immunization with inactivated CA04 and α-GalCer reduces virus in lung and nasal secretions compared to inactivated CA04 alone

**Figure 5:**
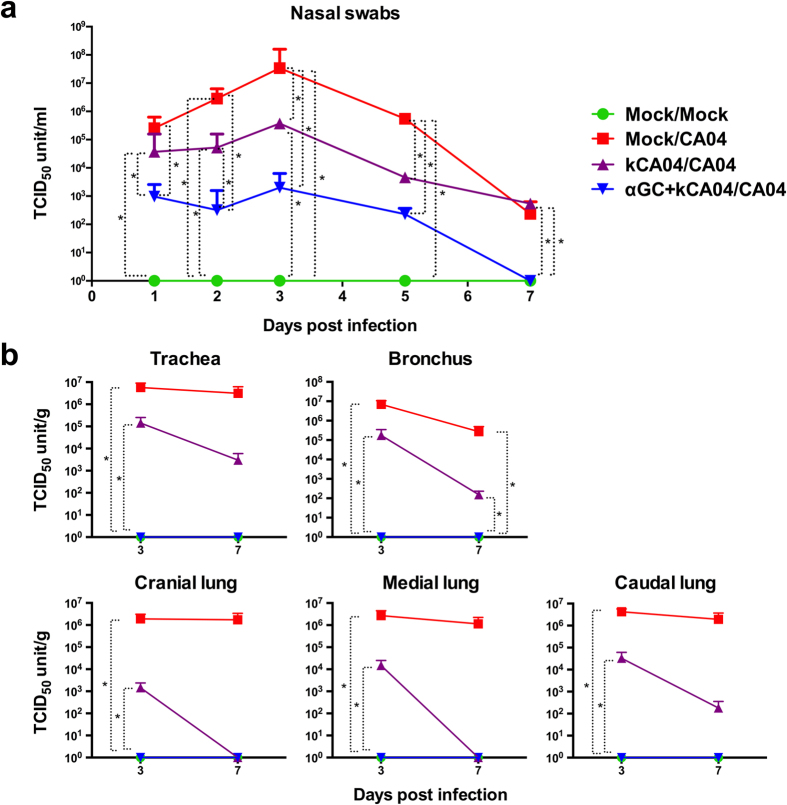
Viral titers in mock-vaccinated pigs (Mock) and pigs intramuscularly vaccinated 2 times with inactivated CA04 H1N1 virus (kCA04) alone or with α-GalCer (αGC) that were mock inoculated or challenged with homologous CA04 virus. Mean virus titers for (**a**) nasal swabs at 1, 2, 3, 5, and 7 days p.i. and (**b**) homogenized airway tissues at 3 and 7 days p.i. Changes in nasal swab titers were analyzed using the SAS PROC MIXED procedure and the Turkey’s test was used to examine treatment differences at each time point for each dependent variable. Changes in tissue titers were analyzed using the Kruskal-Wallis test. *P < 0.05. Data are represented as mean ± SEM.

Nasal swab virus titers were considerably reduced in CA04-challenged pigs immunized with kCA04 and α-GalCer (αGC+kCA04/CA04) compared to pigs that were vaccinated with kCA04 alone (kCA04/CA04), although the differences were not statistically significant on days 3 and 5 p.i. (Fig. 5a). Most strikingly, virus titers were very low (≤10^3^ TCID_50_ units/ml) in αGC+kCA04/CA04 pigs at all time points after inoculation. Vaccination with kCA04 alone (kCA04/CA04) only partially reduced virus titers compared to mock vaccinated and CA04 challenged pigs (Mock/CA04), and only on days 3 and 5 p.i. These collective results show that α-GalCer can significantly decrease virus shedding from nasal fluids when administered with inactivated whole virus compared to pigs immunized with whole virus alone.

Virus titers were also measured in upper and lower airway tissues to determine the effect of the α-GalCer vaccination protocol on viral replication at different locations within the respiratory tract. Virus was undetectable in trachea and bronchus as well as throughout the lung of all αGC+kCA04/CA04 pigs at both 3 and 7 days p.i. However, virus titers were only partially reduced in kCA04/CA04 pigs at 3 days p.i. compared to Mock/CA04 pigs, which represents a time point when high levels of viral replication occurs in the CA04 challenge model (Fig. 5b).

**Figure 6:**
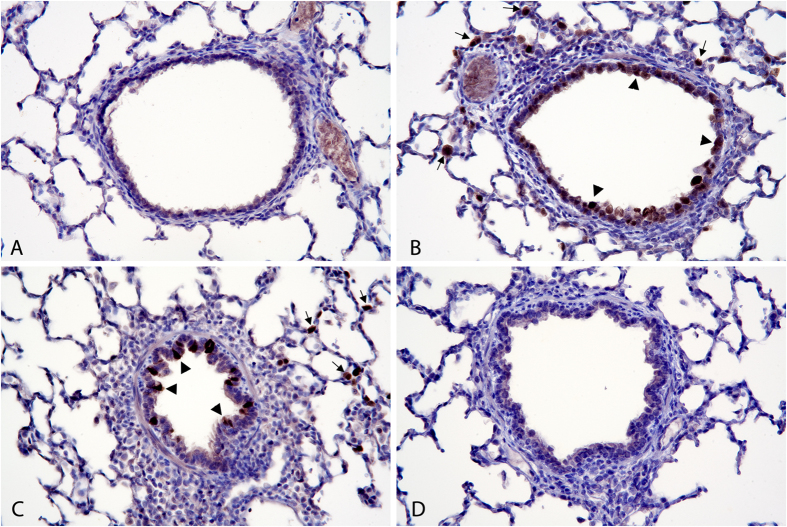
Localization of viral antigen in lung at 3 days post infection. All images are transverse sections of bronchioles and alveolar tissue stained for H1N1 antigen at 174x magnification. Mock/Mock: Image (**A**). Bronchioles and alveolar tissue are negative for H1N1 antigen. Mock/CA04: Image (**B**). There is diffuse staining of H1N1 antigen in bronchiolar epithelium (large arrowheads). Type II alveolar epithelial cells stain multifocally (small arrows). kCA04/CA04: Image (**C**). There is multifocal staining of H1N1 antigen in bronchiolar epithelial cells (large arrowheads). Type II alveolar epithelial cells stain multifocally (small arrows). αGC+kCA04/CA04: Image (**D**). Bronchioles and alveolar tissue are negative for H1N1 antigen.

Consistent with the viral titer results, histopathology performed using a polyclonal antibody against IAV antigen detected diffuse bronchiolar epithelial localization of virus antigen in Mock/CA04 pigs (Fig. 6B & Supplemental Table 5). There was also virus antigen multifocally in type II alveolar epithelial cells in areas of mild interstitial pneumonia surrounding terminal bronchioles. For kCA04/CA04 pigs, multifocal virus antigen was detected in epithelial cells and in type II alveolar epithelial cells in surrounding areas of mild pneumonia (Fig. 6C). In contrast, pigs in the αGC+kCA04/CA04 and Mock/Mock treatment groups did not have detectable virus antigen in bronchiolar epithelial cells or in surrounding type II alveolar epithelial cells (Fig. 6A,D). In addition, they also did not have detectable virus antigen in bronchi. Collectively, these results indicate that using α-GalCer as a vaccine adjuvant in combination with kCA04 elicits immunity that effectively inhibits viral replication and shedding.

## Discussion

In the current study, we demonstrate that α-GalCer co-immunized with inactivated CA04 into the neck muscle of piglets generated protective immunity against homologous influenza infection, indicating that NKT-cell agonists could be used to improve swine vaccine formulations in order to reduce the clinical signs of SI infection and limit the spread of influenza viruses among commercial pigs.

Current commercially available SI vaccines are mostly traditional inactivated whole viruses containing multivalent mixtures of H1 variants and an H3 SI virus subtypes. A major drawback of this approach is that inactivated virus vaccines fail to provide consistent sterilizing immunity that blocks infection, even in years when the vaccine is a good match for circulating viruses^31^. Therefore, research is needed to improve vaccinations for reducing the threat of seasonal and pandemic influenza outbreaks in swine.

Harnessing NKT-cells could be an effective approach to improve inactivated whole-virus vaccines because of their unique ability to kick-start both innate and adaptive immune responses in ways that generate large quantities of antigen-specific antibodies and T cells^32^. NKT-cells share many functions with conventional CD4^+^ T helper cells, including that they license DCs^8^, cross-prime CD8^+^ T cells^22^ and stimulate antibody isotype switching and affinity maturation by B cells^12^,^29^,^30^, which are all important requirements for generating effective protective immunity. However, while the CD4^+^ T cell compartment is comprised of millions of clonotypes that recognize different peptide antigens in an MHC-restricted fashion, NKT-cells interact with the non-polymorphic CD1d molecule and a limited range of glycolipid antigens. This offers the advantage that NKT-cells can be generically activated using synthetic glycolipids for a variety of therapeutic applications. Rodent studies have already demonstrated the potential of NKT-cell agonists as adjuvants for vaccines against influenza^20^,^21^,^22^,^23^,^24^. The current study extends these findings to swine that are an important agricultural species, which are heavily affected by influenza infections and that are capable of transmitting zoonotic influenza strains to humans^33^.

Consistent with what has been reported for mice^21^,^22^,^23^,^24^, we found that vaccinating pigs with α-GalCer and inactivated virus induced immune responses that inhibited viral replication and shedding more effectively than vaccinating pigs with inactivated virus alone. The protective effect of α-GalCer was associated with increased concentrations of anti-hemagglutinin antibodies and a tendency for higher cellular responses against viral antigens. We postulate that the CA04-specific antibodies were most important during early infection for preventing virus particles from entering airway epithelial cells, while cells that did become infected may have been eliminated by cellular responses during the latter stages of disease. It is currently unknown whether the accumulation of  T cells at the intra-epithelial interface between the bronchiolar wall and lumen is important for why viral replication was completely blocked in the α-GalCer vaccinated pigs. It is difficult to assess how much virus clearance was due to T cell-mediated immune responses in the current study. However, it has been shown that α-GalCer co-administration with inactivated IAV promotes the survival of long-term memory cytotoxic T lymphocyte populations capable of boosting cross-protection against heterozygous IAV challenge^22^. Demonstrating the same phenomenon in pigs would be highly relevant for vaccine producers because of the large number of influenza viruses circulating in the field that are constantly changing. Surprisingly, although NKT-cell frequencies in animals receiving α-GalCer varied by more than 200 fold, there was no correlation between systemic NKT-cell concentration and antibody responses in individual pigs. This finding suggests that α-GalCer-mediated humoral immunity depends more on functional subtypes of NKT-cells or the immune cells they *trans*activate rather than the number of NKT-cells *per se* a pig possesses. In contrast, antigen-specific cellular responses were much more correlated to NKT-cell frequency, which is significant because of the importance of T cells for generating long lasting memory and cross-protection against virus infections.

Another similarity to mouse studies was that vaccination with α-GalCer caused an increase of porcine NKT-cells both systemically and within airway tissues. It is possible that some protective immunity provided by the α-GalCer vaccination protocol was partially due to NKT-cells present in lung tissues reducing viral replication through stimulating a variety of early innate immune responses. However, α-GalCer does not protect mice from influenza infections, unless the agonist is co-administered with influenza virus before infection^23^,^24^. This indicates that enhanced adaptive immune responses are likely to be the main reason why α-GalCer+kCA04 vaccinated pigs were better protected compared to pigs that received kCA04 alone. In future, it will be important to treat pigs with α-GalCer alone to definitely address whether NKT-cells confer protection through innate immune mechanisms and/or by stimulating the adaptive immune system.

Our observation that α-GalCer expanded mostly the CD4^−^ subset of NKT-cells may be significant for how swine were protected against disease, because in mice and humans CD4^−^ NKT-cells are highly cytolytic and produce Th1-cytokines^34^, which are important for lysing virus-infected cells. In contrast, the CD4^+^ subset produces both Th1 and Th2 cytokines and has often been associated with tolerogenic activity^35^,^36^,^37^. However, it remains to be determined whether NKT-cell subsets in pigs are functionally equivalent to those in other species.

In conclusion, our study is the first to demonstrate the adjuvanticity of α-GalCer for enhancing inactivated influenza vaccines in pigs. Intramuscular administration of α-GalCer in combination with inactivated virus generated protective immune responses against viral replication within airway tissue, which is of practical importance because most swine vaccines are injected into the neck muscles. The effects of NKT-cell activation we observed in pigs closely mirrors what occurs in mice immunized with α-GalCer and challenged with homologous virus^20^,^21^,^22^,^23^,^24^. This provides encouragement that NKT-cell agonists can also be used for pigs, as they have been for mice, to increase the protective immunity of inactivated whole-virus influenza vaccinations against SI virus infections. Future studies should address whether vaccinating pigs with α-GalCer is capable of enhancing more long-lasting immunity compared to other vaccine adjuvants to be of relevance for commercial pig production. Encouragingly, it has been reported that mice immunized with a single administration of inactivated IAV and α-GalCer were completely protected against a lethal dose of influenza virus infection that lasted for at least 3 months^23^. Experiments should also be performed to establish whether intranasal delivery of α-GalCer induces immune responses in mucosal tissues that can improve the cross-protection of influenza vaccines.

## Methods

### Pigs

Piglets were farrowed from sows that are a mixture of Yorkshire, Chester White, Duroc, Hampshire, and Landrace breeds, which are maintained at the University of Florida’s swine unit. Breeding of sows is performed by artificial insemination using semen collected on-site or from Swine Genetics International (SGI) (Cambridge, IA), Top Cut Genetics (Farmland, IN) or Shaffer’s Goldrush (Albany, IN). Experiments were carried out in accordance with approved guidelines from the United States Department of Agriculture, the National Research Council’s Guide for the Care and Use of Laboratory Animals, as well as all relevant state and federal regulations and policies. The animal care and use committee at the University of Florida approved all experimental protocols under the project number 201308209. Prior to first use, the animals were confirmed free of antibodies to CA04 using hemagglutination inhibition (HAI) assays, and were also checked for the presence of antibodies to influenza H3N2 and B viruses.

### Virus and vaccine preparation

Influenza virus encoding the original consensus sequence of the H1N1pdm09 strain A/California/04/2009 (CA04) was generated by reverse genetics (RG) using plasmids with cloned cDNA encoding all H1N1 viral genes as previously described^38^. Viable virus was recovered in a combination of 293T (human embryonic kidney cells expressing SV40 large tumor antigen) and MDCK (Madin Darby Canine Kidney) cells essentially as described previously^38^, using lipofectamine 2000 (ThermoFisher Scientific, Grand Island, NY) as the transfection agent. The 293T and MDCK were propagated using standard conditions in cell growth media containing low immunoglobulin (Ig) G, heat-inactivated gamma-irradiated fetal bovine serum (FBS) (HyClone Laboratories Inc., Logan, UT). Prior to use, the cells were treated for 3 weeks with plasmocin and verified free of mycoplasma DNA by PCR^39^.

Virus produced from the RG system was subsequently propagated once in MDCK cells incubated at 33 °C, and the virus produced authenticated as CA04 by sequencing of the hemagglutinin and neuraminidase genes. For all virus work, the MDCK cells were in serum-free complete Advanced Dulbecco’s Modified Eagle’s Medium (aDMEM) (ThermoFisher Scientific). The aDMEM was supplemented with 2 mM L-Alanyl-L-Glutamine (GlutaMAX, ThermoFisher Scientific), antibiotics (PSN; 50 μg/mL penicillin, 50 μg/mL streptomycin, 100 μg/mL neomycin) (ThermoFisher Scientific) containing 2.0 μg/ml L-1-tosylamide-2-phenylethyl chloromethyl ketone (TPCK)-treated trypsin, mycoplasma- and extraneous virus-free trypsin (Worthington Biochemical Company, Lakewood, NJ).

Virus titers were calculated from the median (50%) tissue culture infectious dose (TCID_50_), and viral titers expressed as TCID_50_/ml, TCID_50_/g, or their Log_10_-transformed values, as appropriate^40^,^41^. Briefly, the TCID_50_ values were determined by infecting MDCK cells in 96-well microtiter plates with serial dilutions of virus and calculation of the TCID_50_ five days later by the method of Reed and Muench^42^. To produce vaccines, virus particles were suspended in serum-free aDMEM and were UV-inactivated by placing 20 ml aliquots of virus at titers of approximately 3 × 10^7^ TCID_50_/ml in plastic petri dishes and irradiating open dishes in a biosafety cabinet for 30 minutes. Inactivation of each batch of virus was verified using viability checks with 1/20 of each irradiated sample; lack of cytopathic effects in MDCK cells in serum-free media with TPCK-trypsin after 1 week of incubation at 33 °C was accepted as proof of complete inactivation of the virus.

### Preparation of α-GalCer

α-Galactosylceramide was purchased from Avanti Polar Lipids, Inc. (Alabaster, AL) and sonicated in DMSO at 2 mg/ml until fully dissolved. α-GalCer stock solutions were further dissolved in PBS in combination with inactivated virus for administration to pigs.

### Experimental design for vaccination and challenge

Two-week-old piglets were bled from the jugular vein to determine their NKT-cell frequencies in peripheral blood (PB). Twenty-nine piglets were selected and assigned to one of four individual treatments so that each group contained animals with a similar range of NKT-cell frequencies (Table 1). Four days before vaccination, piglets were transferred to isolation rooms. All piglets were seronegative for H1N1 SI viruses in HAI assays. At 3 weeks of age (−32 days post-infection [p.i.]) and 5 weeks of age (−16 days p.i.), pigs in groups 1 and 2 were mock vaccinated intramuscularly (i.m.) with 2 ml of serum-free MEM while those in groups 3 and 4 were respectively injected with 10^6^ TCID_50_ UV-killed CA04 (kCA04) virus alone or in combination with 100 μg/kg of α-GalCer. During the 32 day vaccination period, pigs were bled at the indicated time points to characterize immune cells subsets, including NKT-cell frequencies, and CA04-specific antibody and cellular responses. Sixteen days after the second vaccination (0 days p.i.), pigs in group 1 were mock inoculated intratracheally with virus-free MEM while groups 2 to 4 were inoculated with 10^6^ TCID_50_ CA04 (Table 1). Before inoculation, the animals were sedated with a combination of ketamine (2.2 mg/kg)/xylazine (2.2 mg/kg)/telazol (4.4 mg/kg), administered at a rate of 1 ml/50 lbs of body weight. Virus was delivered using a catheter that was passed through a laryngoscope into the trachea. The intratracheal route of inoculation was selected to assure a consistent level of infection. During the 7 day p.i. period, pigs were monitored daily for disease symptoms such as coughing, nasal and ocular discharge, and weight loss. Blood samples were collected at 2, 5 and 7 days p.i. to measure CA04-specific antibody and cellular responses as well as for analysis of immune cell populations by flow cytometry.

Nasal swabs were collected on 1, 2, 3, 5 and 7 days p.i to measure virus titers. Nasal secretions were collected using sterile flocked swabs (Copan Diagnostics, Inc., Murrieta, CA), and the swabs immersed immediately afterwards in 1 ml of Copan Virus Transport Medium (VTM) in a transport tube. The swab specimens were then frozen at −80 °C until the virus therein was titered on MDCK cells. Antibiotics (penicillin, neomycin, and streptomycin) to control bacteria and fungizone (amphotericin B, from Invitrogen) to control fungi and yeast, was added to the cell-growth media. For virus titrations, the VTM tubes were thawed on ice, then the swabs twirled against the inside surfaces of the tube to extrude collected material into the VTM. The virus titer/ml was obtained, and the value expressed as TCID_50_/swab.

Pigs were euthanized at 3 or 7 days p.i. for necropsy and tissue collection. Euthanasia was accomplished by injecting pigs with 150 mg/kg Pentobarbital Sodium IV through the auricular vein, after pigs were sedated with a combination of ketamine/xylazine/telazol. Death was confirmed by auscultation for lack of heartbeat. Immediately after euthanasia, the axillary vessels in the front legs were severed to exsanguinate the carcass to avoid blood pooling in the lungs. Necropsy was performed by a board-certified veterinary pathologist (WLC). Trachea was bisected and cannulated for fixation of trachea, bronchi and right middle, right caudal and right accessory lung lobes. The left lung was separated at the left main stem bronchus. Fresh samples were aseptically collected from trachea, left bronchus and left lung lobes for analysis of virus titers. Tissue samples were also collected from left lung lobes, right cranial lung lobe, spleen and tracheobronchial lymph nodes (TBLN) for analyzing immune cell populations and CA04-specific cellular responses. Bronchoalveolar lavage fluid (BALF) was collected at necropsy by lavaging the left lung with 50 ml of MEM for examination of immune cell populations as well as viral titers. Results represent the combination of two independent animal studies.

For virus assays, the tissues were weighed and ~0.5 g homogenized in sterile PBS with antibiotics and 0.5% (w/v) purified BSA fraction V to form a 10% (w/v) homogenized suspension using sterile tissue grinders. The homogenates were then titrated and seeded onto confluent MDCK cells that were incubated for five days at 33 °C, and the titer expressed as TCID_50_/g tissue. The lower detection limit was estimated at 20 TCID_50_/g tissue.

### Isolation of leukocytes from blood, spleen, lymph nodes, lung and BALF

Blood samples were collected from the jugular vein in vacutainer plasma tubes coated with sodium heparin (BD Biosciences, San Jose, CA). Isolation of peripheral blood mononuclear cells (PBMCs) was performed using standard procedures with Ficoll-Paque^TM^ PREMIUM (GE Healthcare BioSciences Corp., Uppsala, Sweden) as previously described^25^. Spleen and lymph node tissues were collected and dispersed into single cell suspensions using closed sterile tissue grinders as previously described^25^. Lung tissues were minced using scissors, digested with a mixture of 2 U/ml DNase I (Zymo Research, Irvine, CA) and 250 U/ml collagenase type *III* (Worthington Biochemical, Worthington, NJ) at 37 °C for 45 minutes and filtered through a 100 μm cell strainer. BALF was filtered using a 100 μm cell strainer. An ammonium chloride-based lysis buffer was used to remove residual red blood cells from blood and tissue samples.

### Flow cytometry and antibodies

Immune cell populations in blood, BALF and tissues were characterized using a BD Accuri C6 flow cytometer as previously described^43^. Briefly, single cell suspensions were treated with rat IgG from Sigma-Aldrich (Saint Louis, MO) to block non-specific antibody binding and stained with the indicated fluorochrome-conjugated antibodies at 4 °C for 30 minutes. NKT-cell frequency and subsets were characterized by staining samples with the α-GalCer analog PBS57-loaded mouse CD1d (mCD1d) tetramer reagent or unloaded tetramer provided by the National Institutes of Health Tetramer Core Facility, as well as anti-CD3ε (BB23-8E6-8C8; BD Biosciences, San Jose, CA), and anti-CD4 (74-12-4; Southern Biotech, Birmingham, AL). NKT cells were identified as CD1d tetramer positive cells after gating on single, live, lymphocyte sized CD3^+^ cells. NKT cell subsets were defined according to presence of CD4 as gated on total CD3 positive cells. To assess conventional αβ T cells, γδ T cells, B cells, monocytes and granulocytes, single cell suspensions were stained with antibodies specific for the immune cell surface molecules CD4, CD8 (76-2-11; BD Biosciences), T cell receptor δ (PGBL22A; Kingfisher Biotech, Saint Paul, MN), CD3, CD79α (HM47; BD Biosciences) and CD172α (74-22-15A; BD Biosciences) [43 and Supplemental Fig. 1]. For intracellular FoxP3 staining, cells were surface stained using antibodies for CD4 and CD3 molecules before they were fixed and permeabilized with solutions contained within the eBiosciences FoxP3 staining set (eBiosciences, Inc., San Diego, CA). The recommended manufacturer staining protocol was followed using an anti-mouse/rat FoxP3 antibody (FJK-16s) (Supplemental Fig. 1). Samples collected after SI virus challenge were fixed according to BD the Cytofix/Cytoperm kit from BD Biosciences. Data were analyzed using FlowJo software (Treestar, Palo Alto, CA).

### Serological and mucosal antibody assays

HAI assays were performed essentially as described^44^ using washed 0.5% turkey red blood cells (RBC). Briefly, after serum samples were treated overnight with receptor destroying enzyme (RDE) (Denka Seiken USA, Inc., Campbell, CA) at 37 °C to destroy nonspecific inhibitors of hemagglutination, the sera were heat-treated at 56 °C for 60 min to inactivate remaining RDE activity. They were subsequently treated with a 20% kaolin (Sigma-Aldrich, St. Louis, MO) suspension, and adsorbed with 0.5% turkey red blood cells to remove nonspecific agglutinins. The HAI assays were performed thereafter with 4 hemagglutination units of CA04. The first dilution was 1:10, and 1:2 thereafter.

### IFN-γ enzyme-linked immunosorbent spot assays

The frequencies of CA04-reactive T lymphocytes in PBMC, spleen, lung, and TBLN were quantified using an IFN-γ enzyme-linked immunosorbent spot (ELISPOT) assay as described before^25^. Single cells were suspended in culture medium (RPMI 1640 containing 10% fetal bovine serum and 1% Penicillin/Streptomycin solution) and plated in triplicate at 5 × 10^5^ cells per well into MultiScreen HTS plates (Millipore, Billerica, MA) pre-coated with purified anti-IFN-γ (P2G10, BD Biosciences). Cells were incubated for 3 days at 37 °C with 0 (unstimulated) and 5 × 10^5^ TCID_50_ kCA04 virus particles. After development, spots were read using an automated ELISPOT reader (AID EliSpot High- Resolution Reader System ELHR03). Data are presented as mean number of spots ± SEM/10^4^ leukocytes after subtracting spots counted in unstimulated wells. The mean IFN-γ spots per 10^4^ leukocytes for unstimulated wells were 1.1 for blood, 3.3 for spleen and 0.5 for lung. The number of leukocytes in each well was determined by analyzing each tissue sample by flow cytometry before single cell suspensions were plated. Total live leukocytes were determined based on forward and side scatter and propiduim iodide straining.

### Immunohistochemistry

Right middle and caudal lung lobes that were fixed by intratracheal perfusion at 30 cm H_2_0 pressure with 10% neutral phosphate-buffered formalin for 18–24 hours were blocked out for histopathology and immunohistochemistry. Blocked lung sections were taken from the proximal lobar bronchus, two intermediate level blocks in ventral or ventrocaudal directions. An additional section from ventral and caudal most peripheries was collected for a total of 8 lung sections per pig. Trachea was fixed in formalin. Tissues were dehydrated through alcohol series, cleared in xylene and embedded in paraffin. Paraffin sections were stained with hematoxylin and eosin for histopathologic analysis. Additional paraffin sections for H1N1 antigen or CD3 immunohistochemistry were mounted on charged slides, de-paraffinized and rehydrated prior to heat-induced epitope retrieval in Tris-EDTA, pH 9 at 95 °C for 40 minutes (CD3) or 20 minutes (H1N1 antigen). Endogenous peroxidase activity was quenched by treatment with 0.3% H_2_O_2_ in methanol for 30 minutes. Nonspecific staining was suppressed with Background Punisher (Biocare Medical, Concord, CA) according to the manufacturer protocol. For CD3, sections were treated with rabbit anti-human CD3 (A0452, Dako, Glostrup, Denmark) at 1:100. Mach 4 polymer detection system with horseradish peroxidase (Biocare Medical) was used as described by the manufacturer. Peroxidase activity was detected with Metal Enhanced DAB (3,3′-diaminobenzidine) Substrate Working Solution (Thermo Scientific, Rockford, IL) as described in the kit. For H1N1 antigen detection after epitope retrieval and peroxidase quenching steps, sections were treated with horse blocking serum prior to incubation with goat anti-H1N1 (AB1074, Millipore, Billerica, MA) at 1:500. Bound antibody was detected with an ImmPRESS anti-goat IgG-polymer detection kit (MP-7405, Vector Laboratories, Burlingame, CA) prior to peroxidase activity detection. Slides were lightly stained with Harris hematoxylin.

### Statistical analysis

For NKT-cell frequencies and nasal swab titers, data were analyzed as repeated measures using PROC MIXED of SAS (v9.3, SAS Institute Inc., Cary, NC). The model used was


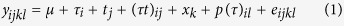


where y_*ijkl*_ = the observation, *μ* = overall mean, *τ*_*i*_ = fixed effect of treatment *i*, *t*_*j*_ = fixed effect of time *j*, (*τt)*_*ij*_ = the interaction between treatment and time, *x*_*k*_ = the random effect of experiment *k*, *p*(*τ*)_*il*_ = the random effect of pig *l* within treatment, and *e*_*ijkl*_ = residual error. Either Toeplitz or spatial power structures were chosen, owing to values of Akaike information criterion (AIC) and the unequal spacing of measurements. Data were log transformed in order to address the heteroscedasticity and non-normally distributed residuals of untransformed data. When the *F*-test for a main effect or interaction was significant (*P* < 0.05), multiple comparisons were done using Tukey’s test.

For other variables, data were analyzed using a Kruskal-Wallis test in SAS using PROC NPAR1WAY and a macro described by Elliot and Hynan^45^. For these variables, residuals were non-normally distributed even after transformation, necessitating the non-parametric test be used. Multiple comparisons were done using Nemenyi’s or Dunn’s test.

Linear regression analyses were preformed using GraphPad Prizm, version 6.0g for Macintosh (GraphPad Software, Inc., La Jolla, CA).

## Additional Information

**How to cite this article**: Artiaga, B. L. *et al*. a-Galactosylceramide protects swine against influenza infection when administered as a vaccine adjuvant. *Sci. Rep*. **6**, 23593; doi: 10.1038/srep23593 (2016).

## Supplementary Material

Supplementary Information

## Figures and Tables

**Table 1:** Characteristics of experimental pigs.

Treatment groups	Number of pigs	Pre-trial NKT cell frequency (% of CD3^+^ PB cells)	Initial body weight (kg)	Average daily gain (kg/day)
Mock/Mock	6	0.23 ± 0.11	7.59 ± 0.60	0.22 ± 0.01
Mock/CA04	8	0.17 ± 0.07	7.73 ± 0.48	0.23 ± 0.02
kCA04/CA04	7	0.23 ± 0.09	7.22 ± 0.65	0.24 ± 0.02
αGC+kCA04/CA04	8	0.29 ± 0.19	8.00 ± 0.58	0.24 ± 0.01

Data were combined from two independent experiments. Values represent mean ± SEM. No significant differences were detected between treatment groups for any of the parameters measured. Experimental groups were: Mock vaccinated and mock challenged (Group 1; Mock/Mock); mock vaccinated and CA04 challenged (Group 2; Mock/CA04), kCA04 vaccinated and CA04 challenged (Group 3; kCA04/CA04); α-GalCer+kCA04 vaccinated and CA04 challenged (Group 4; αGC+kCA04/CA04). Pre-trial NKT cell frequency was analyzed from peripheral blood (PB) at 2 weeks of age.

**Table 2:** Geometric mean HAI titers against CA04 H1N1 in broncho-alveolar lavage fluid (BALF) at 3 and 7 days post-infection.

Treatment groups	3 days p.i.	7 days p.i.
Mock/Mock	<10	<10
Mock/CA04	<10	<10
kCA04/CA04	<10	20
αGC+kCA04/CA04	28.3	33.6

Experimental groups were: Mock vaccinated and mock challenged (Group 1; Mock/Mock); mock vaccinated and CA04 challenged (Group 2; Mock/CA04), kCA04 vaccinated and CA04 challenged (Group 3; kCA04/CA04); α-GalCer+kCA04 vaccinated and CA04 challenged (Group 4; αGC+kCA04/CA04). Geometric mean was calculated as the ‘n’th root product of ‘n’ numbers.
