# Supplementary material for: α-Galactosylceramide protects swine against influenza infection when administered as a vaccine adjuvant
Source: Sci Rep. 2016 Mar 23;6:23593. doi: 10.1038/srep23593 (PMC4804283; doi:10.1038/srep23593)
Supplement: Supplementary Information [file srep23593-s1.pdf]

Supplemental material

α-Galactosylceramide protects swine against influenza infection when administered as a vaccine adjuvant

Bianca L. Artiaga,[a] Guan Yang,[a] Timothy J. Hackmann,[a] Qinfang Liu,[b]* Jürgen A. Richt,[b] Shahram Salek-Ardakani,[c] William L. Castleman,[d] John A. Lednicky,[e,f] and John P. Driver [a]#

Department of Animal Science, University of Florida, Gainesville, FL, USA[a]; Diagnostic Medicine and Pathobiology and Center of Excellence for Emerging and Zoonotic Animal Diseases (CEEZAD), College of Veterinary Medicine, Kansas State University, Manhattan, KS, USA[b]; Department of Pathology, Immunology, and Laboratory Medicine, University of Florida, Gainesville, FL, USA[c]; Department of Infectious Diseases and Pathology, University of Florida, Gainesville, FL, USA[d]; Department of Environmental and Global Health, University of Florida, Gainesville, FL, USA[e]; Emerging Pathogens Institute, University of Florida, Gainesville, FL, USA[f]

Running Head: NKT-cells enhance swine influenza vaccines in pigs

#Address correspondence to John P. Driver, jdriver@ufl.edu.

*Present address: Shanghai Veterinary Research Institute, CAAS, Shanghai, China.

B.L.A. and G.Y. contributed equally to this work.

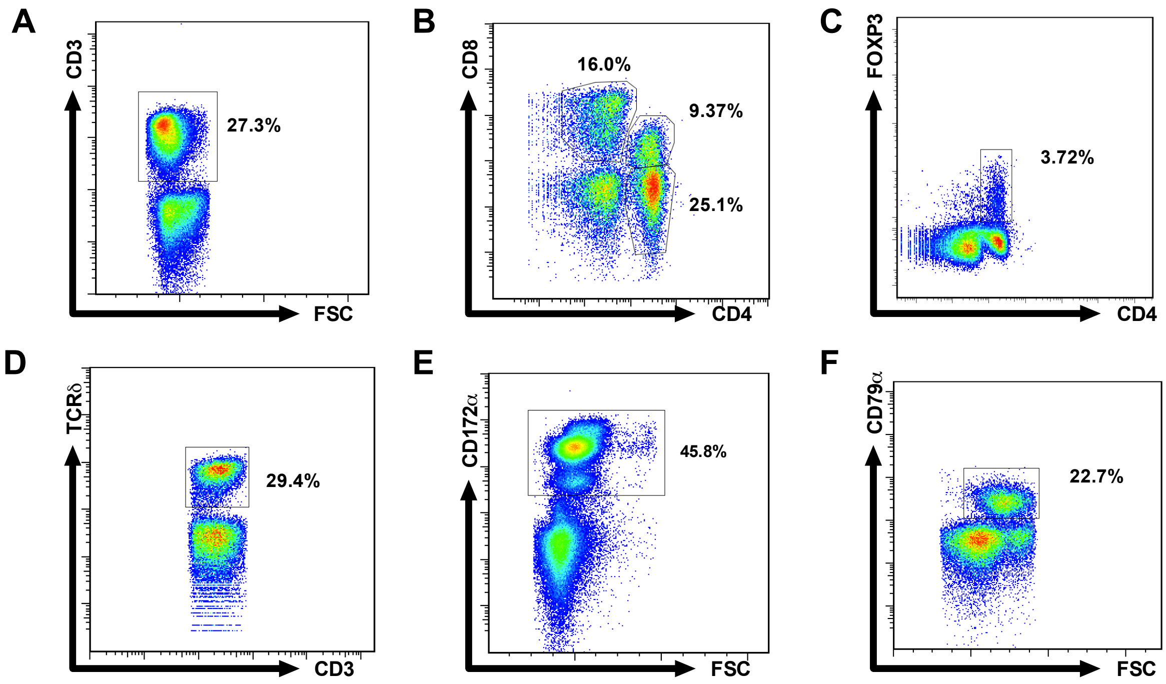

Supplemental Figure S1. Gating strategy to identify immune cell populations in pigs. Single cell suspensions from blood, spleen, tracheobronchial lymph node (TBLN), lungs and bronchoalveolar lavage fluid (BALF) were labeled with mAb cocktails to differentiate (A) $CD3^{+}$ T cells, (B) CD4 and CD8 T cell subsets, (C) $FoxP3^{+}$ regulatory T cells, (D) γδ T cells, (E) monocytes and granulocytes, (F) and B cells. Conventional T cells were identified using anti-CD3 mAb after gating on lymphocyte-sized cells, single cells and live cells. $CD4^{+}$ T cells, $CD8^{+}$ T cells and $CD4^{+}$ $CD8^{+}$ T cells were identified after gating on lymphocyte sized cells, single cells and live cells, as the percentage of $CD3^{+}$ cells that are positive for either one or both markers. Regulatory T cells were identified as a percentage of $CD4^{+}$ T cells that express the transcription factor FOXP3. γδ T cells were identified, after gating on lymphocyte-sized cells, single cells and live cells, as the percentage of $CD3^{+}$ cells positive for TCRδ mAb. Monocytes and granulocytes were identified as CD172α positive cells after gating on single cells and live cells. B cells were identified as CD79α positive cells after gating on lymphocyte-sized cells and single cells. One representative peripheral blood sample is shown.

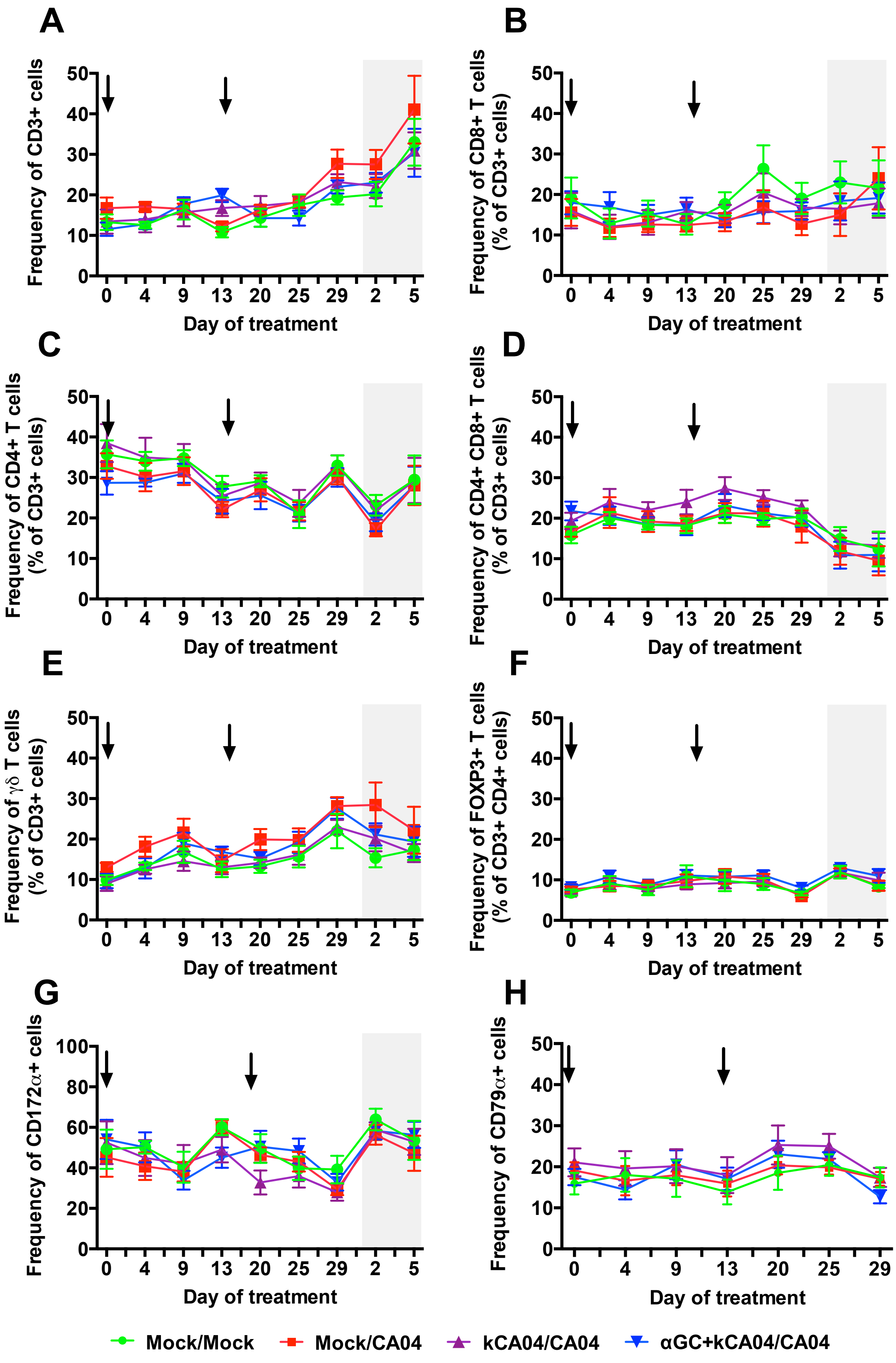
A
Frequency of CD3+ cells
B
Frequency of CD8+ T cells (% of CD3+ cells)
C
Frequency of CD4+ T cells (% of CD3+ cells)
D
Frequency of CD4+ CD8+ T cells (% of CD3+ cells)
E
Frequency of γδ T cells (% of CD3+ cells)
F
Frequency of FOXP3+ T cells (% of CD3+ CD4+ cells)
G
Frequency of CD172α+ cells
H
Frequency of CD79α+ cells
Day of treatment
Mock/Mock
Mock/CA04
kCA04/CA04
αGC+kCA04/CA04

Supplemental Figure S2. Frequency of immune cell populations during the vaccination period and post infection (shaded region). (A) $CD3^+$ cells as a proportion of live lymphocytes, (B) $CD8^+$ T cells, (C) $CD4^+$ T cells, (D) double positive $CD8^+$ $CD4^+$ T cells, (E) $\gamma\delta$ T cells, (F) regulatory T cells, (G) monocytes and granulocytes, (H) B cells (H). Differences in PB immune cell populations were analyzed using the SAS PROC MIXED procedure and no treatment differences were identified. Data are represented as mean ± SEM. Arrows indicate when vaccinations were administered.

Supplemental Table S1. Bronchoalveolar lavage fluid (BALF) leukocyte populations from pigs euthanized on day 3 p.i.

Immune cell population	Mock/Mock	Mock/CA04	kCA04/CA04	αGC+kCA04/CA04
$CD8^+$ cells	20.85 ± 5.30	13.59 ± 4.90	13.33 ± 3.57	15.75 ± 2.65
$CD4^+$ cells	9.01 ± 2.79	5.73 ± 1.68	5.88 ± 1.32	10.07 ± 1.74
$CD4^+$ $CD8^+$ cells	1.70 ± 0.43	1.77 ± 0.48	2.10 ± 1.03	3.06 ± 0.76
$FoxP3^+$ T cells (% of $CD4^+$)	7.52 ± 0.82	9.07 ± 1.80	10.38 ± 1.32	8.85 ± 1.84
$\gamma\delta$ T cells	18.28 ± 2.45	18.15 ± 0.95	15.50 ± 1.51	16.73 ± 2.98
Monocytes and Granulocytes	80.88 ± 6.12	73.93 ± 8.37	86.75 ± 4.86	78.45 ± 3.99

Values represent mean ± SEM for lymphocyte-sized single cells and cell population indicated in parenthesis. Four pigs from each treatment were compared at day 3 post infection. No significant difference ($p > 0.05$) was detected between treatments for any of the immune cell populations tested when analyzed by the Kruskal-Wallis test. Mock vaccinated and mock challenged (Group1; Mock/Mock); mock vaccinated and CA04 challenged (Group 2; Mock/CA04), kCA04 vaccinated and CA04 challenged (Group 3; kCA04/CA04); α-GalCer+kCA04 vaccinated and CA04 challenged (Group 4; αGC+kCA04/CA04).

Supplemental Table S2. Lung leukocyte populations from pigs euthanized on day 3 p.i.

Immune cell population	Mock/Mock	Mock/CA04	kCA04/CA04	αGC+kCA04/CA04
$CD8^+$ cells	23.73 ± 5.34	23.13 ± 6.37	22.28 ± 2.84	23.98 ± 5.75
$CD4^+$ cells	14.61 ± 3.19	11.10 ± 1.73	18.68 ± 2.23	14.17 ± 3.12
$CD4^+$ $CD8^+$ cells	1.34 ± 0.28	2.24 ± 0.86	2.79 ± 1.13	1.82 ± 0.35
$FoxP3^+$ T cells (% of $CD4^+$)	6.75 ± 1.01	6.16 ± 1.09	5.38 ± 0.65	6.36 ± 1.41
$\gamma\delta$ T cells	19.73 ± 1.40	26.90 ± 6.12	20.65 ± 3.88	26.18 ± 4.88
Monocytes and Granulocytes	72.20 ± 3.63	68.25 ± 2.32	67.08 ± 2.96	65.93 ± 4.78

Values represent mean ± SEM for lymphocyte-sized single cells and cell population indicated in parenthesis. Four pigs from each treatment were compared at day 3 post infection. No significant difference ($p > 0.05$) was detected between treatments for any of the immune cell populations tested when analyzed by the Kruskal-Wallis test. Mock vaccinated and mock challenged (Group1; Mock/Mock); mock vaccinated and CA04 challenged (Group 2; Mock/CA04), kCA04 vaccinated and CA04 challenged (Group 3; kCA04/CA04); α-GalCer+kCA04 vaccinated and CA04 challenged (Group 4; αGC+kCA04/CA04).

Supplemental Table S3. Tracheobronchial lymph node (TBLN) leukocyte populations from pigs euthanized on day 3 p.i.

Immune cell population	Mock/Mock	Mock/CA04	kCA04/CA04	αGC+kCA04/CA04
$CD8^+$ cells	15.43 ± 2.04	17.22 ± 4.05	12.19 ± 1.14	15.10 ± 1.35
$CD4^+$ cells	36.13 ± 7.31	35.03 ± 5.27	40.73 ± 7.58	35.23 ± 2.59
$CD4^+$ $CD8^+$ cells	5.55 ± 2.77	7.00 ± 3.77	8.40 ± 4.16	5.76 ± 2.75
$FoxP3^+$ T cells (% of $CD4^+$)	7.60 ± 1.88	6.79 ± 1.04	9.89 ± 0.78	8.90 1.16
γδ T cells	5.48 ± 0.49	5.04 ± 0.64	5.56 ± 0.76	6.43 ± 1.22
Monocytes and Granulocytes	5.58 ± 2.18	9.34 ± 3.78	5.62 ± 1.25	8.55 ± 2.36

Values represent mean ± SEM for lymphocyte-sized single cells and cell population indicated in parenthesis. Four pigs from each treatment were compared at day 3 post infection. No significant difference ($p > 0.05$) was detected between treatments for any of the immune cell populations tested when analyzed by the Kruskal-Wallis test. Mock vaccinated and mock challenged (Group1; Mock/Mock); mock vaccinated and CA04 challenged (Group 2; Mock/CA04), kCA04 vaccinated and CA04 challenged (Group 3; kCA04/CA04); α-GalCer+kCA04 vaccinated and CA04 challenged (Group 4; αGC+kCA04/CA04).

Supplemental Table S4. Splenic leukocyte populations from pigs euthanized on day 3 p.i.

Immune cell population	Mock/Mock	Mock/CA04	kCA04/CA04	αGC+kCA04/CA04
$CD8^+$ cells	15.19 ± 3.12	16.03 ± 2.21	17.60 ± 1.06	20.88 ± 2.08
$CD4^+$ cells	18.85 ± 1.27	15.70 ± 1.44	18.30 ± 1.98	16.18 ± 0.80
$CD4^+$ $CD8^+$ cells	2.47 ± 0.95	3.23 ± 0.85	2.11 ± 0.59	2.97 ± 0.32
$FoxP3^+$ T cells (% of $CD4^+$)	6.90 ± 0.55	6.26 ± 0.71	7.28 ± 0.27	7.48 ± 0.45
$\gamma\delta$ T cells	29.58 ± 2.38	40.63 ± 6.15	35.83 ± 3.45	34.10 ± 5.31
Monocytes and Granulocytes	43.78 ± 3.22	37.08 ± 6.16	32.68 ± 2.91	28.15 ± 3.16

Values represent mean ± SEM for lymphocyte-sized single cells and cell population indicated in parenthesis. Four pigs from each treatment were compared at day 3 post infection. No significant difference ($p > 0.05$) was detected between treatments for any of the immune cell populations tested when analyzed by the Kruskal-Wallis test. Mock vaccinated and mock challenged (Group1; Mock/Mock); mock vaccinated and CA04 challenged (Group 2; Mock/CA04), kCA04 vaccinated and CA04 challenged (Group 3; kCA04/CA04); α-GalCer+kCA04 vaccinated and CA04 challenged (Group 4; αGC+kCA04/CA04).

Supplemental Table S5. Immunohistochemical distribution of H1N1 antigen in bronchial and bronchiolar epithelium and alveolar type II cells

Treatment group	Bronchial Epithelium	Bronchiolar Epithelium	Alveolar type II Cells
Mock/Mock (n=1)	Neg	Neg	Neg
Mock/CA04 (n=2)	++	+++	++
	++	+++	++
kCA04/CA04 (n=2)	++	++	++
	++	++	++
αGC+kCA04/CA04 (n=2)	Neg	Neg	Neg
	Neg	Neg	Neg

Neg = Negative for virus antigen
+ = Rare, focal distribution of virus antigen
++ = Multifocal distribution of virus antigen
+++ = Diffuse distribution of virus antigen
